# Occupational blood exposure beyond needle stick injuries: hospital-based cross-sectional study among healthcare workers in governmental hospitals of Northern Ethiopia

**DOI:** 10.1186/s12913-021-07167-9

**Published:** 2021-10-22

**Authors:** Semere Reda, Mesfin Gebrehiwot, Mistir Lingerew, Awoke Keleb, Tefera chane Mekonnen, Birhanu Wagaye, Amanuel Atamo, Chala Daba, Alelgne Feleke, Metadel Adane

**Affiliations:** 1 Department of Environmental Health, College of Medicine and Health Sciences, Wollo University, Dessie, Ethiopia; 2 Department of Nutrition, School of Public Health, College of Medicine and Health Sciences, Wollo University, Dessie, Ethiopia

**Keywords:** Healthcare workers, Occupational exposure, Infection control, Ethiopia

## Abstract

**Background:**

Occupational blood exposure is one of the major public health problems that healthcare workers (HCWs) are encountering. Most previous occupational blood exposure studies are delimited to needle stick injury, which could underestimate the real level of blood exposure. On the other hand, others deal with crude blood and body-fluids exposure, which possibly overestimate the magnitude of blood exposure. Therefore, this study aimed at determining the prevalence of occupational blood exposure and identifying associated factors among HCWs in the Southern Tigrai zone governmental hospitals of Northern Ethiopia considering all the potential means of blood exposure (needle stick injury, sharp medical equipment injury, and blood splash) while excluding blood-free body-fluids.

**Methods:**

A hospital based cross-sectional study design was employed to gather data from randomly selected HCWs in three governmental hospitals from February to March, 2020. A multivariable logistic regression model was used to identify the independent factors associated with the outcome variable.

**Results:**

From the total of 318 HCWs, 148 (46.5 %) were exposed to blood at least once in their lifetime. Working for more than 40 h per week (AOR= 9.4; 95 % CI: 7.61, 11.41), lack of adequate personal protective equipment (PPE) (AOR=3.88; 95 % CI: 1.64, 5.42), Hepatitis B virus vaccination (AOR=0.54; 95 % CI: 0.12,0.78), recapping used needle sticks (AOR=3.18; 95 % CI: 1.28, 8.83), and lack of infection prevention and patient safety (IPPS) training (AOR=13.5; 95 % CI: 8.12,19.11) were detected to significantly increase the likelihood of occupational blood exposure.

**Conclusions:**

As nearly half of the HCWs were exposed to blood, reducing work load below 40 h per week by employing additional staff members, supplying adequate PPE, avoiding recapping of used needle sticks, and providing IPPS training for the HCWs should be practiced.

**Supplementary Information:**

The online version contains supplementary material available at 10.1186/s12913-021-07167-9.

## Background

Occupational blood exposure is one of the major public health problems that healthcare workers (HCWs) are encountering. Unintended exposure to blood might carry the risk of infection by various blood-borne viruses including Hepatitis B virus (HBV), Hepatitis C virus (HCV), and Human immunodeficiency virus (HIV). No matter how occupational blood exposure is a global issue, the problem is worsening in developing countries in which safety practices and protective devices are not properly utilized [1].

HCWs who experience occupational blood exposure also go through some kinds of psychiatric problems, such as depression and associated post-traumatic stress disorder. The consequences of these effects include absenteeism and poor quality of healthcare service delivery to the community [2].

The World Health Organization (WHO) estimates that among 35 million HCWs worldwide, three million experience occupational blood exposure each year. As a result of these exposures, 150,000 HCWs get infected to HCV, 70,000 to HBV, and 500 to HIV per year. More than 90 % of these infections occurs in developing countries, particularly in sub-Saharan Africa [3]. A systematic review done on the occupational exposures of HCWs to body fluids in 21 African countries showed that the lifetime and one-year blood exposure were 5.7 % and 48 %, respectively [4]. A serious blood-borne infection can cost more than a million dollar in medication, follow up, laboratory testing, clinical evaluation, lost wage, and disability payments. The estimated annual cost due to blood exposure could be 6-7 million dollars in Spain and 4-300 million euros in England. This estimation is based on the reported number of needle stick injuries in different countries and the exact cost still cannot be estimated as large numbers of incidents remain unreported [5].

The Ethiopian federal ministry of health has tried to reform hospitals throughout the country following the “Ethiopian hospitals reform implementation guideline and infection prevention and patient safety strategy” with the prime aim of reducing occupational blood exposure and associated healthcare-acquired infections [6]. Despite the efforts, the prevalence of one-year and lifetime needle stick injuries among the Ethiopian HCWs is as high as 55.1 % and 63.6 %, respectively [7]. Of course, occupational exposures to blood and other body fluids could vary among different regions of the country [8]. The provision of protective equipment, safety trainings and the number of healthcare workforce are indicated to have significant implications for occupational exposures [7, 9]. On top of that, there is poor compliance to infection prevention practices among HCWs [10].

Though there are some studies regarding occupational blood exposure, most of them are delimited to needle stick injury, which could underestimate the real occupational blood exposure burden for HCWs [11, 12]. On the other hand, others deal with crude blood and body-fluids exposure, which possibly overestimate the blood exposure burden [9, 13]. Therefore, considering all means of blood exposure, including needle stick injury, sharp medical equipment injury, and blood splash while excluding blood-free body-fluids will give a better estimate of occupational blood exposure. This study, hence, aimed at assessing the prevalence of occupational blood exposure and associated factors among HCWs in the Southern Tigrai zone governmental hospitals of Northern Ethiopia.

## Materials and methods

### Study design and area

A hospital-based cross-sectional study design was employed from February to March, 2020. The study was conducted in the Southern Tigrai zone of Ethiopia, which is located some 665 km north of the Ethiopian capital, Addis Ababa. Based on the 2019 population projection, Southern Tigrai zone has a total population of 826,042, of whom 404,761 are men and 421,281 are women [14]. According to the Southern Tigrai zone health office report of 2019, there are five governmental hospitals with a total of 834 HCWs.

### Source population, study population, and study unit

All the 834 HCWs in the Southern Tigrai zone governmental hospitals were the source population. The study population includes all the 574 HCWs in the randomly selected Southern Tigrai Zone governmental hospitals. The randomly selected and interviewed HCWs were the study units.

### Inclusion and exclusion criteria

All healthcare professionals (nurses, midwives, physicians, surgeons, laboratory technicians, health officers, anesthetics, dentists, and environmental health officers) and selected supportive staff (i.e., cleaners and laundry workers) who had the chance of direct contact with patients’ blood were included in the study. However, newly recruited and transferred HCWs who did not start their job by any reason were excluded from the study.

### Sample size determination

The sample size was calculated by using single population proportion formula. A 5 % margin of error (d), 95 % confidence level (alpha, α = 0.05 two-tailed), and 28 % proportion were assumed. Accordingly, the total sample size was calculated as:

$$n=\frac{{\left( Z\alpha /2\right)}^2p\left(1-p\right)}{d^2}$$

$$n=\frac{(1.96)^2(0.28)\left(1-0.28\right)}{(0.05)^2}=309$$

Where,

*n*= required sample size.

*Z*= critical value for normal distribution at 95 % confidence level (Z value at α 0.05, two tailed =1.96).

*P*= 28 % prevalence of occupational blood exposure [15].

*d*= 5 % margin of error.

As the study followed two stages of sampling, a design effect of 1.5 was considered. Accordingly, the total sample size was 464 (309 × 1.5). Since sampling was from a finite population (*N*= 834) and as the calculated sample size was more than sufficient to represent the source population, we employed finite population correction. Therefore, the final sample size was calculated as:

*nf* = $$\frac{\text{n}}{1+\text{n}/\text{N}}$$ = 298

Where,

*n* = calculated sample size before sample size correction (464).

*N* = total number of HCWs in the Southern Tigrai zone hospitals (834).

*nf* = final corrected sample size.

By considering a 10 % non-response rate, the total sample size for this study was 328 HCWs.

### Sampling procedure

From the five governmental hospitals in the Southern Tigrai zone, three hospitals (Alamata general hospital, Lemlem Karl general hospital, and Mehoni primary hospital) were selected using lottery method. Then, proportional allocation of samples was made to the selected hospitals based on the total number of HCWs in each hospital. The study units were grouped into seven based on their profession category (i.e., Nurse, Midwifery, Medical doctor, Cleaner, Laboratory, Laundry, and others). Proportional allocation was made again to each profession considering their total number. Accordingly, from each profession, about 57 % study participants were included. This represents about 40 % of the source population. Finally, by using the list of the HCWs from monthly payroll as a sampling frame, simple random sampling technique was used to select 328 study participants.

### Data collection technique

A questionnaire was developed by reviewing pieces of literature of similar studies on occupational blood exposure. First, the questionnaire was prepared in English, and then translated into the local Amharic and Tigrigna languages. The questionnaire has four parts, namely socio-economic and demographic characteristics, institutional characteristics, behavioral and training related characteristics, and blood exposure status information (Appendix A). Data was collected using face to face interview and observation. A HCW was categorized as exposed if he/she had a history of needle stick injury, sharp injury, and/or blood splash in the course of their work [16].

### Data quality assurance

Data quality assurance was in place during questionnaire design, data collection, entry, and analysis. The questionnaire was objective-based, logically sequenced, free of scientific terms, and non-leading. The questionnaire was also pre-tested on 5 % of the sample size (*n*=16) in the neighboring general hospital (Quiha hospital). Based on the pre-test, whenever necessary, the questions were revised, edited, and those found to be unclear or confusing were modified.

Three data collectors and two supervisors were recruited and trained for one day about the study instrument and data collection procedures. Each questionnaire was checked for completeness before winding up the visit to each study participant. Completed questionnaires were checked daily for completeness, legibility, and consistency. Continuous follow-up and supervision were also made by the principal investigator and supervisors throughout the data collection period.

### Data analysis

The collected data was entered into EpiData 3.1 and then exported into SPSS 21.0 for data cleaning and analysis. After cleaning the data for internal consistency, descriptive statistics, such as frequency and percentage were calculated to see the overall distribution of the study variables. In order to identify factors associated with occupational blood exposure, first, bivariable logistic regression analysis with *p* < 0.25 was performed to screen candidate variables. Then, a multivariable analysis was used to control possible confounders. Adjusted odds ratio (AOR) and their 95 % CI were used to measure the association between dependent and independent variables. A significance level of *p* < 0.05 was used to decide the significance of statistical tests. Multi-collinearity among the independent variables was carried out using standard error. As the maximum standard error was 1.95, there was no multicollinearity. The model fitness was checked by Hosmer-Lemeshow test and *p* > 0.05 was considered.

### Ethical considerations

 All methods, including ethical approval and research permission, were performed in accordance with national standards, guaranteeing the anonymity of the respondents. The study was approved by the institutional review board of Wollo University, college of medicine and health sciences (WU/CMHS/1129/02/12). In addition, written consents were obtained from the Southern Tigrai zone coordinator and from the selected hospitals. Besides, respondents were informed about the purpose of the study, and their informed consent was obtained. The respondents’ right to refuse or withdraw from the study was fully maintained and the information provided by each respondent was kept strictly confidential.

## Results

### Socio-economic and demographic characteristics

A total of 328 HCWs have participated in this study. From them, 318 HCWs provided complete information giving a response rate of 97 %. Among the respondents, 193 (60.7 %) were females. The age range of the study subjects was from 20 to 54 years with the mean (±SD) age of 29.4 ± 5.4 years. By profession, 117 (36.8 %) of them were nurses followed by midwives (17 %) and cleaners (14.5 %), respectively (Table 1). Regarding their educational status, more than half (182, 57.2 %) of the HCWs obtained a bachelor degree. Among the study participants, the majority (74.8 %) had five years or less work experience (Table 1).

**Table 1:** Socio-economic and demographic characteristics of HCWs (*N*=318) in the Southern Tigrai zone governmental hospitals, Northern Ethiopia, 2020

Variables	Category	Frequency (n)	Percent (%)
Age (years)	≤25	67	21.1
	26-30	152	47.8
	31-35	68	21.4
	36-40	17	5.3
	> 40	14	4.4
Sex	Male	125	39.3
	Female	193	60.7
Marital status	Single	117	36.8
	Married	165	51.9
	Divorced	29	9.1
	Widowed	7	2.2
Profession	Nurse	117	36.8
	Midwife	54	17.0
	Laboratory	30	9.4
	Medical doctor	33	10.4
	Cleaner	46	14.5
	Laundry	25	7.9
	*Others	13	4.1
Educational status	Below diploma	50	16.0
	Diploma	50	16.0
	BSc degree	182	57.3
	Medical doctor	27	8.5
	Specialty	9	2.2
Work experience (years)	≤ 5	238	74.8
	6-10	61	19.2
	> 10	19	6.0
Monthly income (ETB)	≤ 2000	72	22.6
	2001-3500	32	10.1
	3501-5000	95	29.9
	> 5000	119	37.4

*Others (Anesthesia, Environmental health officer, Radiologist, Psychiatrist, Physiotherapist, Health officer, Maintenance worker, Ophthalmic nurse, and Dentist), ETB-Ethiopian birr

### Institutional characteristics

From the total of 318 HCWs, 180 (56.6 %) were working more than 40 h per week and 142 (44.7 %) had no work time shift. Furthermore, 101 (31.8 %) HCWs had additional responsibilities inside the institution and 92 (28.9 %) were working in private clinics in addition to their routine institutional activities. Unfortunately, about half (49.7 %) of the respondents reported the lack of PPE in the last 12 months and 218 (68.6 %) HCWs have received HBV vaccination. Moreover, 223 (70.1 %) and 42 (13.2 %) had no emergency shower and functional hand washing tap in their working rooms, respectively (Table 2).

**Table 2:** Institutional characteristics of HCWs (*N*=318) in the Southern Tigrai zone governmental hospitals, Northern Ethiopia, 2020

Variables	Category	Frequency (n)	Percent (%)
Level of the hospital	Primary	80	25.2
	General	238	74.8
Working hours per week	≤40	138	43.4
	>40	180	56.6
Working time shift	No	142	44.7
	Yes	176	55.3
Additional responsibilities inside the institution	No	217	68.2
	Yes	101	31.8
Work in private clinics	No	226	71.1
	Yes	92	28.9
^1^Adequate PPE supply in the last 12 months	No	158	49.7
	Yes	160	50.3
Universal safety guideline /protocol in working room	No	67	21.1
	Yes	251	78.9
Presence of functional IPPS committee	No	168	52.8
	Yes	150	47.2
Presence of emergency shower	No	223	70.1
	Yes	95	29.9
Presence of functional hand washing tap	No	42	13.2
	Yes	276	86.8
Three color coded dust bins	No	83	26.1
	Yes	235	73.9
Morning session about occupational blood exposure	No	177	55.7
	Yes	141	44.3
Functional laundry machine	No	192	60.4
	Yes	126	39.6
Adequate safety box in the last 12 months	No	105	33.0
	Yes	140	44.0
HBV vaccination	No	100	31.4
	Yes	218	68.6
Working department	Emergency and injection room	16	5.0
	Pediatrics ward	25	7.9
	Delivery ward	55	17.3
	Medical ward	18	5.7
	Surgical ward	14	4.4
	Operation theater unit	25	7.9
	Cleaner unit	45	14.2
	Laundry unit	23	7.2
	Laboratory unit	24	7.5
	OPD	39	12.3
	*Others	34	10.7

^1^Adequate PPE supply indicates availability of sufficient personal protective equipment whenever required by the healthcare workers, IPPS- Infection prevention and patient safety, *Others (Abortion, MCH, ART, and TB rooms**)**, PPE- personal protective equipment, HBV- Hepatitis B Virus, OPD-outpatient department)

### Behavioral characteristics and training related information

From the total respondents, 144 (37.7 %) HCWs had needle recapping behavior. In addition, 25 (7.9 %), 8 (2.5 %), and 16 (5 %) of the respondents had alcohol-drinking, *Khat* chewing, and cigarette smoking behaviors, respectively. Concerning training experiences, 266 (83.6 %) and 256 (80.5 %) HCWs were not trained about IPPS and healthcare waste management, respectively (Table 3).

**Table 3:** Behavioral characteristics and training related information of HCWs (*N*=318) in the Southern Tigrai zone governmental hospitals, Northern Ethiopia, 2020

Variables	Category	Frequency (n)	Percent (%)
PPE use before any procedure	No	150	47.2
	Yes	168	52.8
Following standard procedures	No	62	19.5
	Yes	256	80.5
Recapping used needle sticks	No	174	45.2
	Yes	144	37.7
Alcohol drinking	No	293	92.1
	Yes	25	7.9
*Khat* chewing	No	310	97.5
	Yes	8	2.5
Cigarette smoking	No	302	95.0
	Yes	16	5.0
^1^Proper waste segregation practice	No	55	17.3
	Yes	263	82.7
Lifetime training on IPPS	No	266	83.6
	Yes	52	16.4
Lifetime training on healthcare waste management	No	256	80.5
	Yes	62	19.5

^1^ proper waste segregation practice was noted whenever waste materials were disposed separately based on the provided color coded dust bins (observation), PPE- Personal protective equipment, IPPS- Infection prevention and patient safety

### Prevalence of occupational blood exposure and related information

In this study, 148 (46.5 %) of the HCWs were exposed to blood at least once in their lifetime (95 % CI: 41 %-52 %). The main route of blood exposure was through blood splash (67.6 %) followed by needle stick injury (36.5 %), and sharp injury (19.6 %). From the exposed HCWs, 47 (31.8 %) of them were exposed at the delivery ward followed by at the operation theater unit (40, 27 %). Moreover, among the exposed respondents, 83 (56.1 %) did not report the exposure to the concerned body (Table 4).

**Table 4:** Occupational blood exposure and related information among blood-exposed HCWs (*N*=148) in the Southern Tigrai zone governmental hospitals, Northern Ethiopia, 2020

Variables	Category		Frequency (n)	Percent (%)
Needle stick injury	No		94	63.5
	Yes		54	36.5
Blood splash	No		48	32.4
	Yes		100	67.6
Sharp injury	No		119	80.4
	Yes		29	19.6
Place of exposure	Emergency and injection	No	119	80.4
		Yes	29	19.6
	Pediatrics ward	No	142	97.2
		Yes	6	2.8
	Delivery ward	No	101	68.2
		Yes	47	31.8
	Medical ward	No	141	95.3
		Yes	7	4.7
	Surgical ward	No	140	94.5
		Yes	8	5.5
	Operation theater unit	No	108	73.0
		Yes	40	27.0
	Cleaner unit	No	120	81.1
		Yes	28	19.9
	Laundry unit	No	137	92.6
		Yes	11	7.4
	Laboratory unit	No	134	90.5
		Yes	14	9.5
	OPD	No	142	95.9
		Yes	6	4.1
	Other working room	No	134	90.5
		Yes	14	9.5
Time of exposure	Day		80	25.2
	Night		55	17.3
	Both		12	3.8
Report of the blood exposure	No		83	56.1
	Yes		65	43.9

OPD-Outpatient department

### Factors associated with occupational blood exposure

While running the bivariable logistic regression analysis, profession, level of the hospital, working hours per week, working time shift, involved in additional responsibilities, adequate PPE supply, safety box supply, needle recapping behavior, vaccination status, proper waste segregation practice, and IPPS training were the candidate variables with *p* < 0.25. These variables were then considered for the multivariate logistic model so as to screen variables that have statistically significant association with blood exposure (Table 5).

In the multivariable logistic regression analysis, working hours per week, lack of PPE supply, needle recapping behavior, vaccination status, and lack of IPPS training showed statistically significant association with occupational blood exposure at *p* < 0.05 (Table 5). The analysis indicated that HCWs who work for more than 40 h per week were 9.42 (AOR= 9.42; 95 % CI: 7.61, 11.41) times more likely to be occupationally exposed to blood than those who work for less than 40 h per week. Similarly, those HCWs who do not have adequate supply of PPE had an odds of 3.88 (AOR=3.88; 95 % CI: 1.64, 5.42) to be occupationally exposed to blood than others. Moreover, HCWs who have needle recapping behavior were 3.18 (AOR=3.18; 95 % CI: 1.28, 8.83) more likely to be exposed to blood as compared with workers who do not recap needle (Table 5). HCWs who do not take HBV vaccination were 46 % less likely to be exposed to blood as compared with their counterparts (AOR=0.54; 95 % CI: 0.12, 0.78). HCWs who do not take IPPS training were also 13.53 more likely to be exposed to blood as compared with the others (AOR=13.53; 95 % CI: 8.12, 19.11) (Table 5).

## Discussion

The aim of this study was to assess occupational blood exposure and associated factors among HCWs in the Southern Tigrai zone governmental hospitals (Northern Ethiopia). The prevalence of lifetime occupational blood exposure was 46.5 %. This estimate was comparable with the report in Tanzania [17] where the prevalence was 48.6 %. In fact, this figure is lower than these reported in Iran (53.4 %), Serbia (60.6 %), Taiwan (71 %), and other part of Ethiopia (Bahirdar) (74 %) [13, 18–20]. On the contrary, our findings revealed a higher prevalence of blood exposure when compared with studies done in Kenya (25 %), India (32.7 %), and Hawassa (Ethiopia) (28 %) [15, 21, 22]. These differences might be subjected to variations in socio-economic, demographic, and cultural characteristics of the study participants, and health facility setups. Exclusion of blood-free body-fluids, in our study, could also contribute to the differences.

In this study, five key risk factors were found to have significant influence on the prevalence of exposure. HCWs who work for more than 40 h per week were more likely (about nine times) to experience blood exposure than others. This finding is consistent with the results obtained in Tehran (Iran), Addis Ababa, and Dessie (Ethiopia) [11, 23, 24]. Reasons might include stress, loss of concentration, and fatigue, which could increase the chance of professional error, risky behaviors, and hence poor compliance with the precautions.

Furthermore, the supply of adequate PPE was identified as an important factor related to occupational blood exposure. In our study, those HCWs who do not have adequate supply of PPE were 3.88 times more likely to be exposed to blood than those who have adequate supply of PPE. This is worrying as 49.73 % of the HCWs reported lack of PPE. Our results are comparable with the findings in Bahir Dar (Ethiopia) [13], which specifically reported that healthcare providers who did not wear PPE were 1.98 times more liable to blood and body fluids exposure than other workers who wore PPE. This is due to the fact that whenever there is lack of PPE supply, HCWs will not have the chance to use them in the healthcare settings while giving any medical care. Consequently, they will become more prone to blood exposure.

**Table 5:** Bivariable and multivariable binary logistic regression analysis results of factors associated with occupational blood exposure among HCWs (*N*=318) in the Southern Tigrai zone governmental hospitals, Northern Ethiopia, 2020

Variables	Category	Exposure status		COR (95 % CI)	p-value	AOR (95 % CI)
		Yes (n)	No (n)			
Profession	Nurse	50	67	2.61 (1.44, 4.21)	0.04*	2.15 (1.12, 4.18)
	Midwife	31	23	3.01 (2.01, 6.51)	0.05*	2.60 (2.30, 5.45)
	Laboratory	18	12	4.02 (3.02, 8.12)	0.03*	3.15 (1.43, 9.60)
	Cleaner	22	24	3.31 (1.33, 6.35)	0.24	1.84 (1.11, 5.16)
	Laundry	13	12	3.91 (1.52, 7.57)	0.20	1.92 (1.28, 4.52)
	Others	2	11	0.81 (2.52, 8.57)	0.25	0.72 (0.25, 0.84)
	Medical doctor	12	21	1		1
Level of the hospital	Primary	44	36	1.57 (1.23, 4.00)	0.05*	1.22 (0.62, 3.34)
	Secondary	104	134	1		1
Working hours per week	≤40 h	26	112	1		1
	>40 h	122	58	9.06 (6.13, 12.52)	0.00*	9.42 (7.61, 11.41)
Working time shift	No	92	84	1.68 (1.15, 4.80)	0.03*	1.54 (1.10, 6.52)
	Yes	56	86	1		1
Additional responsibilities	No	89	128	1		1
	Yes	59	42	2.02 (1.80, 4.82)	0.04*	2.52 (1.85, 6.96)
Adequate PPE supply	No	98	60	3.59 (2.33, 6.55)	0.02*	3.88 (1.64, 5.42)
	Yes	50	110	1		1
Adequate safety box	No	64	41	3.08 (2.22, 5.62)	0.04*	2.52 (1.14, 5.06)
	Yes	47	93	1		1
Needle recapping	No	60	114	1		1
	Yes	88	56	2.98 (2.23, 4.50)	0.01*	3.18 (1.28, 8.83)
HBV vaccination status	No	41	59	0.72 (0.12, 0.82)	0.00*	0.54 (0.12,0.78)
	Yes	107	111	1		1
Proper waste segregation practice	No	33	22	1.93 (1.52, 1.74)	0.23	1.93 (1.41, 6.21)
	Yes	115	148	1		
IPPS training	No	100	25	12.08 (6.12,18.31)	0.05*	13.53 (8.12,19.11)
	Yes	48	145	1		1

^1^reference category, * Significant association (p-value < 0.05) for COR, COR-crude odds ratio, AOR-adjusted odds ratio, PPE- Personal protective equipment, IPPS- Infection prevention and patient safety, HBV- Hepatitis B Virus

Our results also revealed that IPPS training and needle recapping behavior could significantly affect the prevalence of occupational blood exposure. Prevalence was shown to be 13.5 times higher in those who do not attend training courses. Respondents who practice needle recapping had also 318 % more chance to experience exposure than those who do not recap needles after use. This implies the potential of on-job training in enhancing the skills of HCWs, and hence reducing job risks. Other studies conducted in Northeast Ethiopia [11], Southeast Ethiopia [12], Eastern Ethiopia [25], Southwest Ethiopia [26], and South Carolina [27] also reported the importance of IPPS training and needle recapping behavior in influencing occupational exposures.

Moreover, HCWs who take HBV vaccine were 54 % more likely to be exposed to blood as compared with workers who do not take the vaccine. This is possibly associated with negligence and carelessness to avoid blood exposure as HCWs sometimes perceive that vaccination could increase their immunity to HBV. Similar findings were also reported from studies in Gondar (Northern Ethiopia) [9] and Cameroon [28]

## Limitations of the study

This cross-sectional study was based on self-reported lifetime occupational blood exposure, and hence the results were possibly liable to social desirability and recall biases.

## Conclusions

The prevalence of lifetime blood exposure among HCWs in the Southern Tigrai zone governmental hospitals was 46.5 %. The main route of blood exposure was blood splash (67.6 %) followed by needle stick (36.5 %) and sharp (19.6 %) injuries. From the total respondents, 45.2 % had needle recapping behavior and 68.6 % had received HBV vaccination. Working for more than 40 h per week, lack of adequate PPE, HBV vaccination, recapping used needles, and lack of IPPS training were found to significantly increase the likelihood of occupational blood exposure. Therefore, reducing the work load, supplying adequate PPE, avoiding recapping of used needle sticks, and providing IPPS training for HCWs should be emphasized to reduce occupational blood exposure.

## Supplementary information

Additional file 1 Appendix A

## Data Availability

Datasets analyzed during the current study are available from the corresponding author upon reasonable request.

## Abbreviations

AOR: Adjusted odds ratio
ART: Antiretroviral Therapy
COR: Crude odds ratio
ETB: Ethiopian Birr
HCW: Healthcare worker
HBV: Hepatitis B virus
HCV: Hepatitis C virus
HIV: Human immunodeficiency virus
IPPS: Infection prevention and patient safety
MCH: Maternal and child health
OPD: Outpatient department
PPE: Personal protective equipment
TB: Tuberculosis
WHO: World Health Organization
