# Supplementary material for: Occupational blood exposure beyond needle stick injuries: hospital-based cross-sectional study among healthcare workers in governmental hospitals of Northern Ethiopia
Source: BMC Health Serv Res. 2021 Oct 22;21:1136. doi: 10.1186/s12913-021-07167-9 (PMC8532355; doi:10.1186/s12913-021-07167-9)
Supplement: Supplementary file 1 — Appendix A [file 12913_2021_7167_MOESM1_ESM.docx]

**Occupational blood exposure beyond needle stick injuries: hospital-based cross-sectional study among healthcare workers in governmental hospitals of Northern Ethiopia**

Semere Reda^1+^, Mesfin Gebrehiwot^1+^*, Mistir Lingerew^1^, Awoke Keleb^1^, Tefera chane Mekonnen^2^, Birhanu Wagaye^2^, Amanuel Atamo^1^, Chala Daba^1^, Alelgne Feleke^1^, and Metadel Adane^1^

**^1^**Department of Environmental Health, College of Medicine and Health Sciences, Wollo University, Dessie, Ethiopia

**^2^**Department of Nutrition, School of Public Health, College of Medicine and Health Sciences, Wollo University, Dessie, Ethiopia

* Corresponding author: Mesfin Gebrehiwot (PhD)

*Tel.: +251938451295*

E-mail: [gebrehiwotmesfin@yahoo.com](mailto:gebrehiwotmesfin@yahoo.com) (Gebrehiwot, M.)

ORCID: <https://orcid.org/0000-0002-8124-5151>

+Semere Reda and Mesfin Gebrehiwot contributed equally to this work.

**Appendix A.** Questionnaire used to collect data on occupational blood exposure and associated factors among healthcare workers in governmental hospitals of Northern Ethiopia (2020)

Questionnaire ID ___________________ Name of Hospital_________________

Name of the data collector _____________Signature___________ Date ___/__/ 2020

Name of the supervisor ______________Signature __________Date ___/__/2020

| **Part 1**.**Socio-economic and demographic characteristics of healthcare workers (HCWs) in the Southern Tigrai zone governmental hospitals, Northern Ethiopia, 2020** | | | | | | |  |
| --- | --- | --- | --- | --- | --- | --- | --- |
| **S.no** | **Question** | | **Response** | | **Remark** | |  |
| 1. 1 | Age? | | _____years | |  | |  |
| 1. 2 | Sex? | | 1=Male | |  | |  |
|  |  |  | 2=Female | |  |  |  |
|  | Marital status? | | 1=Single | |  | |  |
|  |  |  | 2=Married | |  |  |  |
|  |  |  | 3=Widowed | |  |  |  |
|  |  |  | 4=Divorced | |  |  |  |
| 1. 4 | Profession? | | 1=Nurse | |  | |  |
|  |  |  | 2=Midwife | |  |  |  |
|  |  |  | 3=Laboratory | |  |  |  |
|  |  |  | 4=Medical doctor | |  |  |  |
|  |  |  | 5=Cleaner | |  |  |  |
|  |  |  | 6=Laundry | |  |  |  |
|  |  |  | 7=Other (specify)……… | |  |  |  |
| 1. 5 | Educational status? | | 1=Below diploma | |  | |  |
|  |  |  | 2=Diploma | |  |  |  |
|  |  |  | 3=BSc | |  |  |  |
|  |  |  | 4=MSc | |  |  |  |
|  |  |  | 5=Medical doctor | |  |  |  |
|  |  |  | 6=Specialty | |  |  |  |
|  |  |  | 7=Other (specify)______ | |  |  |  |
| 1. 6 | Work experience? | | ____Years | |  | |  |
| 1. 7 | Monthly income? | | ____Ethiopian birr (ETB) | |  | |  |
| **Part 2. Institutional characteristics of healthcare workers (HCWs) in the Southern Tigrai zone governmental hospitals, Northern Ethiopia, 2020** | | | | | | | |
| **S.no** | | **Question** | | **Response** | | **Remark** | |
|  | | Level of the hospital? | | 1=General | |  | |
|  |  |  |  | 2=primary | |  |  |
|  | | Where is your working department right now? | | 1=Emergency and injection room | |  | |
|  |  |  |  | 2=Pediatrics ward | |  |  |
|  |  |  |  | 3=Delivery ward | |  |  |
|  |  |  |  | 4=Medical ward | |  |  |
|  |  |  |  | 5=Surgical ward | |  |  |
|  |  |  |  | 6=Operation theater unit | |  |  |
|  |  |  |  | 7=Cleaner unit | |  |  |
|  |  |  |  | 8=Laundry unit | |  |  |
|  |  |  |  | 9=Laboratory unit | |  |  |
|  |  |  |  | 10=Outpatient department (OPD) | |  |  |
|  |  |  |  | 11=Others (specify) | |  |  |
|  | | How many working hours do you have per week? | | -------------hours | |  | |
|  | | Do you have working time shift in the hospital? | | 0=No | |  | |
|  |  |  |  | 1=Yes | |  |  |
|  | | Do you have additional responsibilities in this hospital other than your routine work? | | 0=No | |  | |
|  |  |  |  | 1=Yes | |  |  |
|  | | Do you work in private health facilities? | | 0=No | |  | |
|  |  |  |  | 1=Yes | |  |  |
|  | | Did the institution provide adequate personal protective equipment for the last 12 months? | | 0=No | |  | |
|  |  |  |  | 1=Yes | |  |  |
|  | | What type of personal protective equipment is (are) available at this time in the working room? | | 1=Face mask | | **Check by Observation** | |
|  |  |  |  | 2=Eye goggle | |  |  |
|  |  |  |  | 3=Apron | |  |  |
|  |  |  |  | 4=Safety boot | |  |  |
|  |  |  |  | 5=Glove | |  |  |
|  |  |  |  | 6=Cape | |  |  |
|  |  |  |  | 7=Gown | |  |  |
|  |  |  |  | 8=Other(specify) | |  |  |
|  | | Are there any universal safety guidelines/protocols in your working department? | | 0=No | | Observe | |
|  |  |  |  | 1=Yes | |  |  |
|  | | Is there a functional infection prevention and patent safety committee? | | 0=No | | Observe the minute | |
|  |  |  |  | 1=Yes | |  |  |
|  | | Is there a functional hand washing facility in your working room? | | 0=No | | Observe | |
|  |  |  |  | 1=Yes | |  |  |
|  | | Is there a functional emergency shower in your working room? | | 0=No | | Observe | |
|  |  |  |  | 1=Yes | |  |  |
|  | | Are there color coded (black, yellow, and red) waterproof waste storage bins in your working department? | | 0=No | | Observe | |
|  |  |  |  | 1=Yes | |  |  |
|  | | Are there morning sessions about occupational blood exposure in this hospital? | | 0=No | | Look lesson- plan of the session | |
|  |  |  |  | 1=Yes | |  |  |
|  | | Is there a reporting protocol for occupational blood exposure in your institution? | | 0=No | | Observe | |
|  |  |  |  | 1=Yes | |  |  |
|  | | Is there functional laundry machine to wash hospital clothes and sheets? | | 0=No  1=Yes | | Observe & check function | |
|  | | Are there adequate safety boxes within the last 12 months? | | 0=No | | Observe | |
|  |  |  |  | 1=Yes | |  |  |
|  |  |  |  | 3=Not applicable | |  |  |
|  | | If yes for **Q20**, the status of safety box right now? | | 1=overfilled | | Observe | |
|  |  |  |  | 2=<3/4^th^ | |  |  |
|  | | Presence of sharp materials outside the collection box? | | 0=No | | Observe | |
|  |  |  |  | 1=yes | |  |  |
|  | | Are you vaccinated against Hepatitis B virus? | | 0=No | |  | |
|  |  |  |  | 1=yes | |  |  |

| **Part 3 Behavioral and training related characteristics of healthcare workers (HCWs) in the Southern Tigrai zone governmental hospitals, Northern Ethiopia, 2020** | | | |
| --- | --- | --- | --- |
| **S.no** | **Question** | **Response** | **Remark** |
|  | Do you always follow standard procedures? | 0=No |  |
|  |  | 1=yes |  |
|  | Do you always use personal protective equipment (PPE) during procedures? | 0=No | Observe in that moment |
|  |  | 1=Yes |  |
|  | Do you recap used needle sticks? | 0=No |  |
|  |  | 1=yes |  |
|  | Do you drink alcohol and then go to hospital for work? | 0=No |  |
|  |  | 1=Yes |  |
|  | Do you chew *khat* and then go to hospital for work? | 0=No |  |
|  |  | 1=Yes |  |
|  | Do you smoke cigarette and then go to hospital for work? | 0=No |  |
|  |  | 1=Yes |  |
|  | Do you properly segregate waste according to the color-coded dust bin system? | 0=No | Observe the dust bins |
|  |  | 1=Yes |  |
|  | Have you ever been trained on infection prevention and safety practices? | 0=No |  |
|  |  | 1=Yes |  |
|  | Have you ever been trained on healthcare waste management? | 0=No |  |
|  |  | 1=Yes |  |

| **Part 4 Occupational blood exposure and related information among healthcare workers (HCWs) in the Southern Tigrai zone governmental hospitals, Northern Ethiopia, 2020** | | | |
| --- | --- | --- | --- |
| **S.no** | **Question** | **Response** | **Remark** |
| 1 | Have you ever experienced occupational blood exposure? | 0=No | **If yes, proceed to the next questions** |
|  |  | 1=Yes |  |
| 2 | How many times do you exposed to blood? | 1=One time |  |
|  |  | 2=two times  3=three or more times |  |
| 3 | What was the means of exposure? | 1=needle stick injury | More than 1 answer is possible |
|  |  | 2=blood splash and contact |  |
|  |  | 3=sharp injury |  |
| 4 | Where was the working unit while you exposed to blood? | 1=Emergency and injection room |  |
|  |  | 2=Pediatrics ward |  |
|  |  | 3=Delivery ward |  |
|  |  | 4=Surgical ward |  |
|  |  | 5=Medical ward |  |
|  |  | 6=Operation theater unit |  |
|  |  | 7=Cleaner unit |  |
|  |  | 8=Laundry unit |  |
|  |  | 9=Laboratory unit |  |
|  |  | 10=Outpatient department(OPD) |  |
|  |  | 11=Others (specify)____________ |  |
| 5 | At which shift were you exposed? | 1=Day |  |
|  |  | 2=Night |  |
|  |  | 3=Both day and night |  |
| 6 | Did you report the blood exposure? | 0=No |  |
|  |  | 1=Yes |  |
